# Association between *MCU* Gene Polymorphisms with Obesity: Findings from the *All of Us* Research Program

**DOI:** 10.3390/genes15040512

**Published:** 2024-04-19

**Authors:** Jade Avery, Tennille Leak-Johnson, Sharon C. Francis

**Affiliations:** 1 Department of Physiology, Morehouse School of Medicine, Atlanta, GA 30310, USA; javery@msm.edu; 2 Institute of Translational Genomic Medicine, Morehouse School of Medicine, Atlanta, GA 30310, USA; 3 Cardiovascular Research Institute, Morehouse School of Medicine, Atlanta, GA 30310, USA

**Keywords:** SNP, *MCU*, obesity, All of Us

## Abstract

Obesity is a public health crisis, and its prevalence disproportionately affects African Americans in the United States. Dysregulation of organelle calcium homeostasis is associated with obesity. The mitochondrial calcium uniporter (*MCU*) complex is primarily responsible for mitochondrial calcium homeostasis. Obesity is a multifactorial disease in which genetic underpinnings such as single-nucleotide polymorphisms (SNPs) may contribute to disease progression. The objective of this study was to identify genetic variations of *MCU* with anthropometric measurements and obesity in the All of Us Research Program. Methods: We used an additive genetic model to assess the association between obesity traits (body mass index (BMI), waist and hip circumference) and selected *MCU* SNPs in 19,325 participants (3221 normal weight and 16,104 obese). Eleven common *MCU* SNPs with a minor allele frequency ≥ 5% were used for analysis. Results: We observed three *MCU* SNPs in self-reported Black/African American (B/AA) men, and six *MCU* SNPs in B/AA women associated with increased risk of obesity, whereas six *MCU* SNPs in White men, and nine *MCU* SNPs in White women were protective against obesity development. Conclusions: This study found associations of *MCU* SNPs with obesity, providing evidence of a potential predictor of obesity susceptibility in B/AA adults.

## 1. Introduction

According to 2017–2018 data from the National Health and Nutrition Examination Survey, more than 76 million American adults, which represents 42.8% of the population, are obese [1]. If present trends continue, by 2030 78% of American adults are projected to be overweight or obese [2]. Obesity is defined as the accumulation of excessive fat resulting from energy imbalance. Furthermore, obesity perpetrates a financial burden of USD 150–210 billion annually in associated medical costs, with the medical cost for people who have obesity being USD 1429 higher than those of normal weight [3,4]. According to the Centers for Disease Control (CDC), there are disparities in obesity burden across racial and ethnic groups, with the highest prevalence of obesity in non-Hispanic Black adults (38.4%) overall, followed by Hispanic adults (32.6%) and non-Hispanic White adults (28.6%) [3,4]. Furthermore, obesity is a highly polygenic disease in which genetic variations such as single-nucleotide polymorphisms (SNPs) may contribute to an individual’s development of this disease. Previous studies have established a significant association between several genes and obesity, such as the fat mass and obesity-associated (*FTO*) gene, melanocortin-4 receptor (*MC4R*) gene, and leptin receptor (*LEPR*) gene [5,6,7,8]. The public health, financial burden, and health inequity associated with obesity underpin the importance of elucidating the genetic basis of this polygenic disease.

The All of Us (AoU) Research Program, funded by the National Institutes of Health (NIH), is a robust research tool that enables the assessment of biological, clinical, social, and environmental determinants of health and disease while simultaneously reflecting diversity in the United States [9]. The primary aim of the AoU Research Program is to understand the association between biomarkers and genes and disease risk [10]. Earlier studies implicated the role of mitochondrial dysfunction in the pathogenesis of obesity [11]. Mitochondria are dynamic organelles responsible for energy production, calcium homeostasis, and cell death. The mitochondrial calcium uniporter (*MCU*) is a multimeric complex that regulates calcium influx into the mitochondrial matrix [12]. Calcium is a vital internal messenger that regulates energy metabolism [13]. Several studies previously demonstrated that obesity leads to cytosolic and/or organelle calcium flux dysregulation [14]. A critical gap exists in our understanding of the potential role of *MCU* genetic variants in obesity susceptibility. Studies of *MCU* allelic variants and their association with obesity are not fully understood. Findings from this study contribute to understanding the link between the *MCU* gene and obesity susceptibility. Given this significant gap in the literature, we aimed to elucidate the genetic etiology of eleven *MCU* SNPs with anthropometric traits and obesity in self-reported White and B/AA subsamples of the AoU Research Program. Moreover, associations found in this study could hold promise for finding novel therapeutic targets. This is of particular importance in the current era of increased use of weight loss management medications [15].

## 2. Materials and Methods

### 2.1. AoU Participant Cohort Construction

This study was performed on cross-sectional and genomic data generated from AoU participant data collected as previously described [16] using the AoU Researcher Workbench, a cloud computing platform. This cloud-based platform allows authorized researchers to annotate groups of participants data based on specific inclusion and/or exclusion criteria (Cohort Builder), create datasets for analysis using the health information of the cohorts (Dataset Builder), and use Jupyter Notebooks to save datasets using high-powered queries Python 3 and R programming languages. Our cohort and dataset construction and code/R commands used for analysis are in the Research Workbench workspace ‘*MCU* Genetic Variation, Obesity, and Vascular Disease’ and are available to authorized United States researchers at the AoU research workbench (https://www.researchallofus.org/data-tools/workbench/ accessed on 1 January 2024) or from the corresponding author upon reasonable request. 

We utilized the Dataset Builder to create participant datasets categorized into two groups: those with normal weight (body mass index (BMI): 20–24.9) and those classified as obese (BMI: 30+), determined through observation of Systemized Nomenclature of Medicine–Clinical Terminology (SNOMED-CT) codes [17]. Our inclusion criteria encompassed self-reported Black/African American (B/AA) and White participants, aged 18–80, with a recorded sex at birth of either female or male, and identified as not Hispanic or Latino ethnicity. Also, participants were not pregnant.

### 2.2. Measurement of Biochemical and Anthropometric Traits 

Each participant’s clinic visit consisted of biospecimen collection (blood, urine, saliva) and physical measurements, including physiologic (blood pressure and heart rate), and anthropometric measurements (height, weight, waist circumference, and hip circumference) [9]. BMI uses weight (kilograms)/height (meters squared) and is a widely accepted indicator of adiposity. Anthropometric measurements, waist and hip circumference, were obtained using standardized protocols from the phenotypes and exposures (PhenX) toolkit [18]. These measurements are also indicators of abdominal obesity. Biochemical traits were measured in the biospecimen samples collected for total cholesterol, low-density lipoprotein (LDL), high-density lipoprotein (HDL), triglycerides, and hemoglobin A1c (HbA1c) levels. 

### 2.3. SNP Selection and Genotyping

Participants who consented to participate in the AoU research program donated fresh whole blood as a primary source of DNA [9]. Subsequently, this DNA was sent to genome centers for genomic analysis, including whole genome sequencing (WGS) and genotyping. Details on biospecimen collection and processing, genome center sample receipt, WGS library construction, sequencing, array genotyping, and genomic data curation are previously described [9]. The candidate gene approach was used to select genetic variants in the *MCU* gene located on chromosome 10q22.1 and GRCh38/hg38 position base-pair coordinates chr10:72696113-72851477. Employing dbSNP, we curated SNPs based on their minor allele frequency (MAF) within African American (AA) populations. Specifically, we focused on SNPs showing an intermediate frequency, falling between the MAFs observed in the native ancestral populations of European and African descent. For this study, 11 SNPs were selected with an MAF ≥ 5% for further analyses. The MAFs for the analyzed SNPs ranged from 5.2% to 52.9%. The Hardy Weinberg equilibrium (HWE) was assessed to check data quality using chi-square statistics at a significance level of <10^−4^. 

### 2.4. Statistical Analysis (Descriptive Statistics and Regression Analysis)

To summarize the study variables, mean ± standard deviation (SD) was used to present the descriptive statistics of the study population. For this study, we examined the first physiologic, physical, and anthropometric measurement logged for each participant. Outliers and missing values were removed using distribution-based outlier detection. The Tukey statistical test was used to analyze differences between multiple groups in GraphPad Prism 9.4 [19]. For association analyses between *MCU* SNPs and normal continuous outcomes (BMI, waist circumference, hip circumference) and categorical outcomes (obesity), we used multivariate linear and logistic regression under an a priori additive model using age and HbA1c levels as covariates for model adjustments. We adjusted for HbA1c as studies have widely established that obesity and type II diabetes are comorbidities in American adults [20]. The beta (β) values were used to determine the direction of effect for each MCU SNP. 

## 3. Results

### 3.1. Clinical Characteristics of the Study Population

The clinical characteristics of the study population stratified by self-reported race, BMI, and sex at birth are summarized in Table 1. A total of 19,325 participants (3221 normal weight and 16,104 obese) were included in this study from the AoU cohort, of whom 77.9% (12,546/16,104) were female and 22.1% (3558/16,104) were male Anthropometric measurements and biochemical characteristics were compared by weight status across sex at birth in White participants (Table 1) and in B/AA participants (Table 1). Additionally, clinical characteristics were compared by weight status across self-reported race stratified by sex at birth (Table 1). The anthropometric measurements (weight, waist circumference, hip circumference, and BMI) were significantly higher in subjects with obesity than subjects with normal weight independent of self-reported race and sex at birth (Table 1). HDL levels were statistically lower in obese individuals compared to the normal weight participants for each study group (Table 1). 

Sex-specific differences in weight, waist circumference, hip circumference, BMI, total cholesterol, LDL, HDL, triglycerides, HbA1c levels, and systolic blood pressure (SBP) of White study participants were observed, with White obese women having higher values of hip circumference, total cholesterol, LDL, and HDL levels relative to White obese men (Table 1). Moreover, White men with obesity had higher weight, triglycerides, waist circumference, and SBP levels relative to White obese women (Table 1). Sex-specific differences in weight, waist circumference, hip circumference, BMI, total cholesterol, LDL, HDL, triglycerides, HbA1c levels, diastolic, and systolic blood pressure of B/AA study participants were observed with B/AA obese women having higher values for hip circumference, total cholesterol, LDL, and HDL levels relative to B/AA obese men (Table 1). Obese B/AA men had higher weight, waist circumference, triglycerides, SBP, and HbA1c levels relative to B/AA obese women (Table 1).

### 3.2. Association of MCU SNPs with Obesity in the AoU Cohort

Association analyses revealed no significant associations between the 11 *MCU* SNPs and increased obesity in the White study participants, suggesting decreased risk for obesity (Table 2). SNPs rs2121094, rs7092031, rs2121097, rs7081970, rs3009550, and rs3009554 were significantly associated with decreased risk of obesity in both White men and women. Interestingly, rs6415912, rs6480644, and rs9416029 showed an association with a decreased probability of obesity development. 

Three *MCU* SNPs in B/AA men revealed evidence of association with increased susceptibility to obesity: rs34072881 (OR: 1.30; 95% CI: 1.12–1.50; *p*-value: 0.0004), rs3009554 (OR: 1.68; 95% CI: 1.53–1.86; *p* < 0.0001), and rs9416029 (OR: 1.46; 95% CI: 1.33–1.59; *p* < 0.0001). In the B/AA women, 6 *MCU* SNPs rs3009556 (OR: 1.06; 95% CI: 1.00–1.11; *p*-value: 0.035), rs6415912 (OR: 1.07; 95% CI: 1.02–1.12; *p*-value: 0.007), rs6480644 (OR: 1.07; 95% CI: 1.01–1.12; *p*-value: 0.011), rs9416029 (OR: 1.60; 95% CI: 1.48–1.73; *p* < 0.0001), rs3009550 (OR: 1.08; 95% CI: 1.05–1.12; *p* < 0.0001), and rs3009554 (OR: 1.38; 95% CI: 1.27–1.50; *p* < 0.0001) was significantly associated with obesity, indicating an increased probability of developing obesity (Table 2). The SNPs rs9416029 (B/AA men: OR = 1.46; B/AA women: OR = 1.60) and rs3009554 (B/AA men: OR = 1.68; B/AA women: OR = 1.38) were significantly associated with increased risk of obesity in both B/AA men and women. 

### 3.3. Association of MCU SNPs with BMI in the AoU Cohort

The association of 11 *MCU* SNPs with BMI was also explored in the AoU cohort (Table S3). Multiple linear regression models identified that the variants were not associated with BMI (*p*-values range: 0.099–0.955). Interestingly, in White men and women, all *MCU* SNPs revealed lower BMI (β range: −0.026–−0.059). Conversely, in B/AAs, 9 *MCU* SNPs (rs34072881, rs6415912, rs6480644, rs2121094, rs7092031, rs2121097, rs7081970, rs9416029, and rs3009554) were observed with higher BMI (β range: 0.003–0.063) (Table S3). 

### 3.4. Association of MCU SNPs with Waist Circumference in the AoU Cohort

As shown in Table 3, only 1 SNP, rs3009554, is associated with increased waist circumference (β = 0.244; *p* = 0.026). We observed differences in β coefficients with regard to self-reported race. Five *MCU* SNPs (rs34072881, rs6415912, rs9416029, rs3009550, and rs3009554) were observed with decreased waist circumference (β range: −0.030–−0.062) in White participants; however, these SNPs were observed with increased waist circumference (β range: 0.006–0.244) in B/AA study participants (Table S4). In both White men and women, *MCU* SNPs rs3009556, rs6480644, rs2121094, rs7092031, rs2121097, and rs7081970 were observed with decreased waist circumference (β range: −0.003–−0.050) (Table S4). Differences in β with respect to sex at birth were observed in B/AA participants. Interestingly, rs3009556, rs6480644, rs2121097, and rs7081970 revealed increased waist circumference in B/AA men (β range 0.009–0.078) and decreased waist circumference (β range: −0.001–−0.040) in B/AA women (Table S4). 

### 3.5. Association of MCU SNPs with Hip Circumference in the AoU Cohort

Association analyses between *MCU* SNPs and hip circumference revealed 4 SNPs (rs6415912, rs6480644, rs2121094, and rs7092031) significantly associated with decreased hip circumference in B/AA men (Table 3). Nominal associations were observed with rs2121097 and rs7081940 and decreased hip circumference in B/AA men (*p*-value = 0.052; Table 3). In contrast, these 6 SNPs were observed with an increased hip circumference in B/AA women (β range: 0.031–0.049; Table 3 and Table S5). 

In White participants, SNPs rs3009556, rs6480644, and rs6415912, and in B/AA participants rs34072881, rs9416029, and rs3009554 were observed with increased hip circumference (β range: 0.002–0.121; Table S5). Furthermore, seven SNPs (rs2121094, rs7092031, rs2121097, rs7081970, rs9416029, rs3009550, and rs3009554) were observed with decreased hip circumference (β range: −0.007–−0.021) in the White participants (Table S5). 

## 4. Discussion

This is the first analysis of the *MCU* gene with obesity traits in two racially diverse cohorts from the AoU Research Program. We explored cross-sectional analyses to determine the relationship between eleven polymorphisms in the *MCU* gene with anthropometric measurements and obesity risk in White and B/AA participants.

Obesity is a polygenic disease in which the genetic basis of body weight regulation is complex [21]. Our interest in the *MCU* gene was due to the established link between obesity and dysregulated organelle calcium influx and the important role of *MCU* in the regulation of mitochondrial calcium homeostasis [14]. Associations found in this study are significant in determining genetic risk factors of obesity predisposition in a diverse cohort of study participants. 

Our study revealed *MCU* SNPs were significantly associated with increased waist circumference and decreased hip circumference in B/AA men of the AoU cohort. We found 3 *MCU* SNPs in B/AA men: rs34072881 (OR: 1.30; 95% CI: 1.12–1.50), rs3009554 (OR: 1.68; 95% CI: 1.53–1.86), and rs9416029 (OR: 1.46; 95% CI: 1.33–1.59) significantly associated with obesity, increasing the probability of developing obesity. Obesity is a major risk for hypertension [22]. B/AA participants were more hypertensive relative to the White men and women. These significant associations between *MCU* SNPs and obesity in B/AA men could be due to their hypertensive state. Further studies are needed to elucidate the underlying mechanisms.

As there are no reported functional consequences for the *MCU* SNPs that increased susceptibility to obesity in B/AA men and women, we used the online tool, HaploReg v4.2 [23], to predict potential regulatory motifs that may be impacted by these variants. HaploReg revealed that seven significant risk variants (rs6415912, rs6480644, rs3009550, rs34072881, rs3009554, rs9416029, and rs3009556) may impact multiple transcription factor binding motifs. These motifs include those associated with PITX2, RREB1, DMRT5, GATA3, TAL1, HNF, NR4A, and SREBP, which act as transcriptional activators. Additionally, the analysis identified motifs associated with the transcriptional repressor FOXP1, as well as the dual function repressor/activator motifs of RREB1 and MAF [24]. Interestingly, based on the LOD (Logarithm of Odds) values for the reference and alternate allele, it appears the variant alleles might increase the binding affinity of transcriptional activators and decrease the binding affinity of the transcriptional repressors. Based on these functional motif alterations, we speculate that the risk alleles identified in B/AA men and women may upregulate *MCU* gene expression, leading to an increase in mitochondrial Ca^2+^ uptake. Indeed, it has been reported that pharmacological and genetic disruption of *MCU* in mice ameliorated diet-induced obesity [25]. Moreover, high levels of *MCU* were observed in the visceral adipose tissue (VAT) of mice fed a high-fat diet and in obese human subjects with and without type 2 diabetes. In the rodent studies, high levels of *MCU* in VAT were associated with enhanced mitochondrial Ca^2+^ uptake [26]. 

This study found that the minor alleles were either protective or associated with an increased probability of developing obesity. In nine *MCU* SNPs, the minor alleles were protective against obesity in White women, with OR between 0.86 and 0.94. Whereas six of the eleven *MCU* SNPs: rs3009556 (OR: 1.06; *p*-value = 0.035), rs6415912 (OR: 1.07; *p*-value = 0.007), rs6480644 (OR: 1.07; *p*-value = 0.011), rs9416029 (OR: 1.60; *p* < 0.0001), rs3009550 (OR: 1.0), and rs3009554 (OR: 1.38) were significantly associated with obesity in B/AA women. These findings suggest that these *MCU* SNPs may be a potential determinant factor for developing obesity in B/AA women. For 6 *MCU* SNPs, the minor alleles are protective against obesity in White men, with OR between 0.80 and 0.90. No previous studies, to our knowledge, have reported these associations.

B/AA women have the highest prevalence of obesity in the United States [27]. *MCU* SNPs within the enhancer regulatory region, rs3009550 and rs6415912, were significantly associated with obesity in B/AA women in the AoU cohort [28,29]. *MCU* SNPs in the enhancer region may result in upregulation of *MCU* gene expression. Previous studies demonstrate *MCU* stimulates fat differentiation and fat deposition in rodent and human adipose tissue [25,26]. Excessive accumulation of adipose tissue characterizes obesity. Thus, *MCU* SNPs may activate the enhancer/promoter region, further leading to increased *MCU* gene expression and inducing white fat deposition. Furthermore, metabolic alteration in adipocytes leads to the secretion of dysregulated adipokines that promote obesity development [30]. Increased *MCU* gene expression and/or activity alteration could be a potential clinical biomarker to show a propensity for obesity development in B/AA women. 

However, our analyses did not reveal a significant association between *MCU* SNPs and higher BMI in any of the study population. Remarkably, reduced β values for *MCU* SNPs were shown in White men and women and were associated with lower BMI and decreased waist circumference, whereas increased β values for *MCU* SNPs observed in B/AA men and women were associated with higher BMI. Furthermore, increased β values were observed with increased hip circumference in B/AA women. These findings could potentially be due to epigenetics such as environmental exposures (stress, smoking, lifestyle, etc.). Further studies are needed to elucidate the molecular mechanisms of these observed differences. 

This work suggests that mutation in the *MCU* gene is a causative factor for obesity. This strong association between *MCU* and obesity could potentially be due to the role of *MCU* in mitochondrial oxidative metabolism and insulin resistance [31]. Previous studies have shown that imbalanced mitochondrial oxidative metabolism results in obesity and diabetes [32]. *MCU* allows calcium entry into the mitochondrial matrix, which promotes mitochondrial oxidative metabolism. Moreover, *MCU* plays an essential role in glucose-induced insulin secretion [31]. Furthermore, previous studies have demonstrated that altered mitochondrial calcium flux in adipocytes may play a role in lipid accumulation and obesity [25,26]. Thus, alterations in *MCU* function can impair mitochondrial oxidative metabolism, lipid metabolism, and insulin secretion, which are prominent features of obesity. 

After Bonferroni correction, one would consider a *p*-value ≤ 0.0045 as significant evidence for association. After Bonferroni correction, SNPs rs9416029, rs3009550, and rs3009554 in B/AA women and SNPs rs9416029, rs3009554, and rs34072881 in B/AA men remained significant. 

There are glaring health disparities in the quality of obesity care that disproportionately affect racial and ethnic minorities, specifically B/AA [33]. Genetic ancestry is defined as the genetic information we inherit from our ancestors [34]. This testing has been useful in assessing predicted disease risk. The *MCU* SNP, rs34072881, is a synonymous variant; strikingly, in this study, we found that this SNP was exclusively present in the B/AA participants. The reported allele frequencies in dbSNP are T = 0.1947 in Africans and T = 0.00042 in Europeans. This strongly suggests that rs34072881 of the *MCU* gene is inherited in those with higher African ancestry. However, these findings require further confirmation in other cohorts with balanced stratification of White and B/AA study participants.

Genetic variation in *MCU* exerts opposing effects of genetic risk of obesity development in the White and B/AA study populations. The molecular mechanisms underlying this population-specific phenomenon are unclear. One explanation for the heterogeneity of the *MCU* SNPs could be differences in their environmental and behavioral factors. Previous studies have observed that ethnic differences influenced genetic determinants of obesity originating from specific associations with environmental factors such as cigarette smoking, physical activity, alcohol consumption, time spent sitting, and total energy intake [35]. Gene–environment interactions are widely explored in obesity and may represent a plausible mechanism to explain this complex phenomenon [35,36]. Another potential explanation is the distinct genetic admixture prevalent in African Americans across the United States. Several studies have indicated a harmonious relationship between genetic admixture and environmental factors as an explanation of observed ethnic differences in obesity-related traits [37,38].

While the findings in this study are insightful, we recognize there are limitations such as the lack of existing literature on transcriptional regulation of *MCU* and the effect of *MCU* genetic variants on regulation of *MCU* function. These limitations make it difficult to precisely know how the identified SNPs may be altering *MCU* expression and/or function to increase susceptibility or protection from obesity. Thus, studies examining *MCU* transcriptional regulation are important areas for further exploration. Additionally, having more of a balance in the stratification of the population between the normal weight and obese study groups could potentially assist with identifying more associations between SNPs and obesity. Relying solely on BMI as a measure of adiposity without considering body fat mass composition might mask underlying associations. Additionally, we did not account for the potential confounding effects of genetic admixture. 

## 5. Conclusions

In this study, we found a novel association between *MCU* genetic variants with obesity in adults from the AoU Research Program. Specifically, we observed that the minor alleles, i.e., allele G of rs9416029, allele A of rs3009554, and allele T of rs34072881, were associated with obesity in B/AA men. Additionally, our study revealed that allele G of rs9416029, allele C of rs3009556, allele G of rs6415912, allele T of rs6480044, allele A of rs3009550, and allele A of rs3009554 are associated with obesity in B/AA women. These findings suggest that these *MCU* SNPs may play a significant role in the development of obesity in B/AA adults.

## Figures and Tables

**Table 1:** Clinical characteristics of the study population.

Variables	Normal Weight (BMI 20–24.9)				Obese (BMI 30+)			
	White Men	White Women	B/AA Men	B/AA Women	White Men	White Women	B/AA Men	B/AA Women
	(*n* = 744)	(*n* = 1369)	(*n* = 493)	(*n* = 615)	(*n* = 4113)	(*n* = 6532)	(*n* = 1429)	(*n* = 4030)
Age (years)	62.87 ± 13.74 ^A^	60.41 ± 13.77	59.29 ± 11.78 ^E^	56.86 ± 13.46	64.28 ± 11.52 ^B^	60.42 ± 12.39	59.39 ± 10.5 ^F^	57.77 ± 12.08
Weight (kg)	76.85 ± 10.65 ^A,C^ (*n* = 702)	63.81 ± 9.98 ^D^ (*n* = 1337)	73.7 ± 13.28 ^E,G^ (*n* = 469)	66 ± 13.12 ^H^ (*n* = 590)	106.02 ± 15.42 ^B^ (*n* = 3944)	91.51 ± 15.98 (*n* = 6355)	102.84 ± 17.79 ^F^ (*n* = 1356)	92.84 ± 17.69 (*n* = 3868)
BMI (kg/m^2^)	24.39 ± 2.9 ^A,C^ (*n* = 677)	23.48 ± 2.86 ^D^ (*n* = 1271)	23.78 ± 3.02 ^G^ (*n* = 469)	24.04 ± 3.38 ^H^ (*n* = 540)	33.8 ± 4.16 ^B^ (*n* = 3878)	34.09 ± 4.84 (*n* = 6078)	33.4 ± 4.6 ^F^ (*n* = 1376)	34.33 ± 5.15 (*n* = 3782)
WC (cm)	91.9 ± 8.9 ^A,C^ (*n* = 614)	81.4 ± 9.77 ^D^ (*n* = 1246)	87.53 ± 9.08 ^E,G^ (*n* = 459)	84.85 ± 10.48 ^H^ (*n* = 568)	115.13 ± 11.3 ^B^ (*n* = 3400)	105.11 ± 12.52 (*n* = 5747)	111.98 ± 12.56 ^F^ (*n* = 1310)	105.87 ± 12.7 (*n* = 3828)
HC (cm)	99.28 ± 6.35 ^C^ (*n* = 618)	98.89 ± 6.91 ^D^ (*n* = 1230)	97.75 ± 6.91 ^G^ (*n* = 443)	98.82 ± 7.64 ^H^ (*n* = 542)	115.23 ± 9.35 ^B^ (*n* = 3417)	119.73 ± 10.88 (*n* = 5694)	115.39 ± 10.16 ^F^ (*n* = 1318)	119.79 ± 11.34 (*n* = 3763)
LDL (mg/dL)	96.64 ± 34.53 ^A,C^ (*n* = 384)	106.37 ± 32.44 ^D^ (*n* = 716)	95.3 ± 33.2 ^G^ (*n* = 257)	101.84 ± 33.26 ^H^ (*n* = 303)	104.23 ± 35.4 ^B^ (*n* = 2448)	111.02 ± 32.58 (*n* = 3495)	104.88 ± 34.8 (*n* = 771)	108.29 ± 32.86 (*n* = 2069)
HDL (mg/dL)	47.23 ± 15.43 ^A,C^ (*n* = 539)	60.29 ± 16.68 ^D^ (*n* = 992)	51.87 ± 17.05 ^E,G^ (*n* = 384)	57.12 ± 16.61 ^H^ (*n* = 473)	41.45 ± 10.71 ^B^ (*n* = 3382)	50.73 ± 13.05 (*n* = 4812)	44.43 ± 12.99 ^F^ (*n* = 1129)	50.96 ± 13.19 (*n* = 3121)
TG (mg/dL)	112.74 ± 54.91 ^A,C^ (*n* = 524)	101 ± 47.96 ^D^ (*n* = 987)	104.14 ± 51.39 ^G^ (*n* = 379)	102.02 ± 49.89 (*n* = 469)	147.47 ± 64.01 ^B^ (*n* = 3117)	130.34 ± 60.62 (*n* = 4785)	122.99 ± 62.26 ^F^ (*n* = 1072)	109.42 ± 55.01 (*n* = 3153)
HbA1c (%)	5.61 ± 0.53 ^C^ (*n* = 374)	5.5 ± 0.49 ^D^ (*n* = 737)	5.57 ± 0.54 ^G^ (*n* = 255)	5.71 ± 0.54 ^H^ (*n* = 317)	5.87 ± 0.7 ^B^ (*n* = 2633)	5.74 ± 0.66 (*n* = 3700)	6.01 ± 0.73 (*n* = 771)	5.93 ± 0.67 (*n* = 2199)
DBP (mm/Hg)	86.77 ± 21.86 (*n* = 208)	90.54 ± 25.96 (*n* = 281)	109.72 ± 31.92 (*n* = 54)	115.02 ± 28.54 (*n* = 54)	91.91 ± 23.23 ^B^ (*n* = 823)	88.15 ± 23.7 (*n* = 1523)	105.75 ± 29.05 (*n* = 125)	106.58 ± 27.34 (*n* = 309)
SBP (mm/Hg)	125.09 ± 26.39 ^A^ (*n* = 207)	113.09 ± 29.75 ^D^ (*n* = 278)	100.75 ± 27.42 (*n* = 55)	88.89 ± 21.01 ^H^ (*n* = 57)	125.66 ± 31.37 (*n* = 827)	121.83 ± 28.65 (*n* = 1544)	114.96 ± 29.83 (*n* = 130)	108.06 ± 32.95 (*n* = 320)

Data presented as mean ± SD and statistical comparisons between different study groups based on sex at birth and weight status. Comparisons include (A) normal weight White men vs. White women *p* < 0.05, (B) obese White men vs. White women *p* < 0.05, (C) normal weight White men vs. obese White men *p* < 0.05, and (D) normal weight White women vs. obese White women *p* < 0.05, (E) normal weight B/AA men vs. B/AA women *p* < 0.05, (F) obese B/AA men vs. B/AA women *p* < 0.05, (G) normal weight B/AA men vs. obese B/AA men *p* < 0.05, and (H) normal weight B/AA women vs. obese B/AA women *p* < 0.05. Abbreviations: BMI: body mass index; kg: kilograms; m: meters; cm: centimeters; mg: milligrams; dL: deciliters; mm: millimeters; Hg: mercury; WC: waist circumference; HC: hip circumference; TG: triglycerides; DBP: diastolic blood pressure; and SBP: systolic blood pressure.

**Table 2:** Association of *MCU* SNPs with obesity in the AoU cohort.

SNP	Alleles	White Men		White Women		B/AA Men		B/AA Women	
		OR (95% CI)	*p*-Value	OR (95% CI)	*p*-Value	OR (95% CI)	*p*-Value	OR (95% CI)	*p*-Value
rs34072881	C/T	--	--	--	--	1.30 (1.12–1.50)	0.0004 *	0.77 (0.69–0.86)	<0.0001 *
rs3009556	T/C	--	--	--	--	0.75 (0.70–0.80)	<0.0001 *	1.06 (1.00–1.11)	0.035 *
rs6415912	A/G	--	--	0.94 (0.91–0.98)	0.002 *	0.79 (0.74–0.84)	<0.0001 *	1.07 (1.02–1.12)	0.007 *
rs6480644	C/T	--	--	0.94 (0.90–0.97)	0.0004 *	0.79 (0.74–0.84)	<0.0001 *	1.07 (1.01–1.12)	0.011 *
rs2121094	G/A	0.89 (0.84–0.95)	0.0002 *	0.89 (0.86–0.92)	<0.0001 *	0.74 (0.69–0.79)	<0.0001 *	--	--
rs7092031	G/T	0.89 (0.84–0.95)	0.0002 *	0.88 (0.85–0.92)	<0.0001 *	0.70 (0.66–0.75)	<0.0001 *	--	--
rs2121097	A/T	0.89 (0.84–0.95)	0.0002 *	0.89 (0.85–0.92)	<0.0001 *	0.70 (0.66–0.75)	<0.0001 *	--	--
rs7081970	C/T	0.80 (0.75–0.85)	<0.0001 *	0.87 (0.84–0.90)	<0.0001 *	0.72 (0.67–0.76)	<0.0001 *	--	--
rs9416029	A/G	--	--	0.89 (0.86–0.92)	<0.0001 *	1.46 (1.33–1.59)	<0.0001 *	1.60 (1.48–1.73)	<0.0001 *
rs3009550	G/A	0.80 (0.75–0.85)	<0.0001 *	0.86 (0.83–0.90)	<0.0001 *	0.93 (0.90–0.97)	0.0006 *	1.08 (1.05–1.12)	<0.0001 *
rs3009554	G/A	0.90 (0.85–0.95)	0.0004 *	0.89 (0.86–0.92)	<0.0001 *	1.68 (1.53–1.86)	<0.0001 *	1.38 (1.27–1.50)	<0.0001 *

Abbreviations: SNP: single nucleotide polymorphism; CI: confidence interval. The odds ratios and corresponding *p*-values were calculated using logistic regression adjusting for age and HbA1c levels. *p* was significant at a value of <0.05. * Statistical significance.

**Table 3:** Association of *MCU* SNPs with waist and hip circumference from the AoU cohort.

Obesity Trait	SNP	Alleles	White Men		White Women		B/AA Men		B/AA Women	
			Estimate (β)	*p*-Value	Estimate (β)	*p*-Value	Estimate (β)	*p*-Value	Estimate (β)	*p*-Value
WC	rs3009554	G/A	−0.062 (0.052)	0.238	−0.046 (0.041)	0.269	0.244 (0.108)	0.026 *	0.139 (0.116)	0.233
HC	rs6415912	A/G	0.041 (0.040)	0.307	0.022 (0.029)	0.442	−0.111 (0.054)	0.042 *	0.047 (0.052)	0.365
HC	rs6480644	C/T	0.041 (0.040)	0.307	0.024 (0.029)	0.405	−0.111 (0.054)	0.042 *	0.049 (0.052)	0.342
HC	rs2121094	G/A	−0.007 (0.040)	0.865	−0.019 (0.029)	0.513	−0.109 (0.053)	0.040 *	0.049 (0.052)	0.472
HC	rs7092031	G/T	−0.007 (0.040)	0.865	−0.021 (0.029)	0.472	−0.109 (0.053)	0.040 *	0.031 (0.051)	0.549
HC	rs2121097	A/T	−0.007 (0.040)	0.865	−0.021 (0.029)	0.472	−0.103 (0.053)	0.052	0.045 (0.051)	0.377
HC	rs7081970	C/T	−0.007 (0.040)	0.865	−0.020 (0.028)	0.493	−0.103 (0.053)	0.052	0.045 (0.051)	0.377

Abbreviations: SNP: single nucleotide polymorphism; WC: waist circumference; HC: hip circumference. A linear regression was performed with an additive model; waist and hip circumference for adjusted age and HbA1c levels. The associations’ results are estimate, standard error (β), and the corresponding *p*-value. *p* was significant at a value of <0.05. * Statistical significance.

## Data Availability

The data supporting this study’s findings are available from the corresponding author upon reasonable request.

## Supplementary Materials

The following supporting information can be downloaded at: https://www.mdpi.com/article/10.3390/genes15040512/s1, Table S1: Frequency of Selected MCU SNPs in the AoU cohort; Table S2: Association of MCU SNPs with obesity in the AoU cohort; Table S3: Association of MCU SNPs with BMI in the AoU cohort; Table S4: Association of MCU SNPs with waist circumference in the AoU cohort; Table S5: Association of MCU SNPs with hip circumference in the AoU cohort.
