# Supplementary material for: Association between MCU Gene Polymorphisms with Obesity: Findings from the All of Us Research Program
Source: Genes (Basel). 2024 Apr 19;15(4):512. doi: 10.3390/genes15040512 (PMC11050077; doi:10.3390/genes15040512)
Supplement: Supplementary file 1 [file genes-15-00512-s001.zip › genes-2947002-supplementary.pdf]

Supplementary Materials

Table S1: Frequency of Selected MCU SNPs in the AoU cohort

SNPs	A1	A2	MAF (%)	Type of Mutation
rs34072881	C	T	5.2%	synonymous
rs3009556	T	C	72.4%	Intronic
rs6415912	A	G	70.9%	Intronic
rs6480644	C	T	70.8%	Intronic
rs2121094	G	A	66.7%	Intronic
rs7092031	G	T	66.7%	Intronic
rs2121097	A	T	66.6%	Intronic
rs7081970	C	T	66.5%	Intronic
rs9416029	A	G	53.8%	Intronic
rs3009550	G	A	53.4%	Intronic
rs3009554	G	A	52.9%	Intronic

Abbreviations: SNP: single-nucleotide polymorphism; MAF: minor allele frequency.

Table S2: Association of MCU SNPs with obesity in the AoU cohort

SNP	Alleles	White Men		White Women		Black/AA Men		Black/AA Women	
		**OR (95% CI)**	**P-value**	**OR (95% CI)**	**P-value**	**OR (95% CI)**	**P-value**	**OR (95% CI)**	**P-value**
rs34072881	C/T	--	--	--	--	1.30(1.12-1.50)	0.0004*	0.77(0.69-0.86)	<0.0001*
rs3009556	T/C	0.98(0.93-1.04)	0.505	1.00(0.97-1.04)	0.799	0.75(0.70-0.80)	<0.0001*	1.06(1.00-1.11)	0.035
rs6415912	A/G	1.05(0.99-1.11)	0.0868	0.94(0.91-0.98)	0.002*	0.79(0.74-0.84)	<0.0001*	1.07(1.02-1.12)	0.007
rs6480644	C/T	1.05(0.99-1.11)	0.0887	0.94(0.90-0.97)	0.0004*	0.79(0.74-0.84)	<0.0001*	1.07(1.01-1.12)	0.011
rs2121094	G/A	0.89(0.84-0.95)	0.0002*	0.89(0.86-0.92)	<0.0001*	0.74(0.69-0.79)	<0.0001*	0.98(0.93-1.03)	0.403
rs7092031	G/T	0.89(0.84-0.95)	0.0002*	0.88(0.85-0.92)	<0.0001*	0.70(0.66-0.75)	<0.0001*	0.98(0.93-1.03)	0.365
rs2121097	A/T	0.89(0.84-0.95)	0.0002*	0.89(0.85-0.92)	<0.0001*	0.70(0.66-0.75)	<0.0001*	1.00(0.95-1.04)	0.872
rs7081970	C/T	0.80(0.75-0.85)	<0.0001*	0.87(0.84-0.90)	<0.0001*	0.72(0.67-0.76)	<0.0001*	1.00(0.95-1.05)	0.87
rs9416029	A/G	1.00(0.95-1.05)	0.977	0.89(0.86-0.92)	<0.0001*	1.46(1.33-1.59)	<0.0001*	1.60(1.48-1.73)	<0.0001*
rs3009550	G/A	0.80(0.75-0.85)	<0.0001*	0.86(0.83-0.90)	<0.0001*	0.93(0.90-0.97)	0.0006*	1.08(1.05-1.12)	<0.0001*
rs3009554	G/A	0.90(0.85-0.95)	0.0004*	0.89(0.86-0.92)	<0.0001*	1.68(1.53-1.86)	<0.0001*	1.38(1.27-1.50)	<0.0001*

Abbreviations: SNP: single nucleotide polymorphism; MAF: minor allele frequency; CI: confidence interval. The odds ratios and corresponding p-values were calculated using logistic regression adjusting for age and HbA1c levels. p was significant at a value of <0.05. * Statistical significance.

Table S3: Association of MCU SNPs with BMI in the AoU cohort

SNP	Alleles	White Men		White Women		Black/AA Men		Black/AA Women	
		**Estimate(β)**	**P-value**	**Estimate(β)**	**P-value**	**Estimate(β)**	**P-value**	**Estimate(β)**	**P-value**
rs34072881	C/T	--	--	--	--	0.037(0.107)	0.729	0.063(0.106)	0.554
rs3009556	T/C	-0.045(0.035)	0.206	-0.038(0.024)	0.117	0.045(0.045)	0.318	-0.012(0.047)	0.805
rs6415912	A/G	-0.027(0.036)	0.455	-0.027(0.025)	0.278	0.030(0.044)	0.494	0.026(0.048)	0.589
rs6480644	C/T	-0.027(0.036)	0.455	-0.026(0.025)	0.293	0.030(0.044)	0.494	0.023(0.047)	0.629
rs2121094	G/A	-0.043(0.035)	0.219	-0.039(0.025)	0.117	0.007(0.043)	0.865	0.007(0.046)	0.874
rs7092031	G/T	-0.043(0.035)	0.219	-0.040(0.025)	0.109	0.007(0.043)	0.865	0.006(0.046)	0.903
rs2121097	A/T	-0.040(0.035)	0.219	-0.040(0.025)	0.109	0.011(0.043)	0.792	0.003(0.046)	0.955
rs7081970	C/T	-0.043(0.035)	0.219	-0.038(0.025)	0.124	0.007(0.043)	0.874	0.003(0.047)	0.955
rs9416029	A/G	-0.059(0.036)	0.099	-0.038(0.025)	0.125	0.014(0.053)	0.794	0.025(0.067)	0.713
rs3009550	G/A	-0.038(0.030)	0.208	-0.032(0.021)	0.13	0.039(0.027)	0.16	-0.020(0.030)	0.494
rs3009554	G/A	-0.059(0.036)	0.099	-0.038(0.025)	0.125	0.062(0.059)	0.291	0.058(0.072)	0.429

Abbreviations: BMI: body mass index; SNP: single nucleotide polymorphism; MAF: minor allele frequency. A linear regression was performed with an additive model; BMI for adjusted age and HbA1c levels. The associations' results are estimate, standard error (β), and the corresponding p-value. p was significant at a value of <0.05. * Statistical significance

Tables S4: Association of MCU SNPs with waist circumference in the AoU cohort

SNP	Alleles	White Men		White Women		Black/AA Men		Black/AA Women	
		**Estimate(β)**	**P-value**	**Estimate(β)**	**P-value**	**Estimate(β)**	**P-value**	**Estimate(β)**	**P-value**
rs34072881	C/T	--	--	--	--	0.149(0.144)	0.302	0.135(0.175)	0.442
rs3009556	T/C	-0.026(0.052)	0.615	-0.033(0.041)	0.42	0.078(0.071)	0.274	-0.007(0.075)	0.928
rs6415912	A/G	-0.030(0.052)	0.566	-0.004(0.042)	0.928	0.016(0.070)	0.815	0.006(0.075)	0.934
rs6480644	C/T	-0.030(0.052)	0.566	-0.003(0.042)	0.942	0.016(0.070)	0.815	-0.001(0.075)	0.992
rs2121094	G/A	-0.032(0.051)	0.528	-0.050(0.041)	0.233	-0.002(0.069)	0.98	-0.025(0.074)	0.733
rs7092031	G/T	-0.032(0.051)	0.528	-0.050(0.041)	0.226	-0.002(0.069)	0.98	-0.019(0.074)	0.802
rs2121097	A/T	-0.032(0.051)	0.528	-0.050(0.041)	0.223	0.009(0.069)	0.895	-0.040(0.073)	0.586
rs7081970	C/T	-0.032(0.051)	0.528	-0.049(0.041)	0.241	0.018(0.069)	0.798	-0.040(0.073)	0.586
rs9416029	A/G	-0.062(0.052)	0.238	-0.046(0.041)	0.269	0.132(0.098)	0.181	0.154(0.108)	0.155
rs3009550	G/A	-0.039(0.044)	0.382	-0.064(0.035)	0.072	0.011(0.045)	0.804	0.035(0.048)	0.467
rs3009554	G/A	-0.062(0.052)	0.238	-0.046(0.041)	0.269	0.244(0.108)	0.026*	0.139(0.116)	0.233

Abbreviations: SNP: single nucleotide polymorphism; MAF: minor allele frequency. A linear regression was performed with an additive model; waist circumference for adjusted age and HbA1c levels. The associations' results are estimate, standard error (β), and the corresponding p-value. p was significant at a value of <0.05. * Statistical significance.

Table S5: Association of MCU SNPs with hip circumference in the AoU cohort

SNP	Alleles	White Men		White Women		Black/AA Men		Black/AA Women	
		**Estimate(β)**	**P-value**	**Estimate(β)**	**P-value**	**Estimate(β)**	**P-value**	**Estimate(β)**	**P-value**
rs34072881	C/T	--	--	--	--	0.091(0.130)	0.487	0.028(0.123)	0.822
rs3009556	T/C	0.002(0.040)	0.961	0.004(0.029)	0.902	-0.071(0.055)	0.199	-0.005(0.052)	0.92
rs6415912	A/G	0.041(0.040)	0.307	0.022(0.029)	0.442	-0.111(0.054)	0.042*	0.047(0.052)	0.365
rs6480644	C/T	0.041(0.040)	0.307	0.024(0.029)	0.405	-0.111(0.054)	0.042*	0.049(0.052)	0.342
rs2121094	G/A	-0.007(0.040)	0.865	-0.019(0.029)	0.513	-0.109(0.053)	0.040*	0.049(0.052)	0.472
rs7092031	G/T	-0.007(0.040)	0.865	-0.021(0.029)	0.472	-0.109(0.053)	0.040*	0.031(0.051)	0.549
rs2121097	A/T	-0.007(0.040)	0.865	-0.021(0.029)	0.472	-0.103(0.053)	0.052	0.045(0.051)	0.377
rs7081970	C/T	-0.007(0.040)	0.865	-0.020(0.028)	0.493	-0.103(0.053)	0.052	0.045(0.051)	0.377
rs9416029	A/G	-0.011(0.040)	0.793	-0.021(0.029)	0.473	0.059(0.073)	0.419	0.083(0.068)	0.218
rs3009550	G/A	0.021(0.034)	0.544	-0.023(0.025)	0.352	-0.052(0.034)	0.133	-0.010(0.033)	0.769
rs3009554	G/A	-0.011(0.040)	0.793	-0.021(0.029)	0.473	0.121(0.078)	0.122	0.070(0.072)	0.331

Abbreviations: SNP: single nucleotide polymorphism; MAF: minor allele frequency. A linear regression was performed with an additive model; hip circumference for adjusted age and HbA1c levels. The associations' results are estimate, standard error (β), and the corresponding p-value. p was significant at a value of <0.05. * Statistical significance.
